# Gut Microbiota Regulates Brain–Bone Axis to Influence Osteoporosis Pathogenesis and Treatment

**DOI:** 10.34133/research.1178

**Published:** 2026-03-16

**Authors:** Haojun Shi, Lei Huang, John H. Zhang, Chengwan Shen, Nan Zhang, Cui Lv, Litao Shao, Mengyao Li, Zijin Sun, Liang Shi, Gongchang Yu, Yisheng Chen

**Affiliations:** ^1^ Faculty of Chinese Medicine and State Key Laboratory of Quality Research in Chinese Medicines, Macau University of Science and Technology, Macau, Macau SAR, China.; ^2^ Department of Molecular Cell and Cancer Biology, University of Massachusetts Chan Medical School, Lake Avenue North, Worcester, MA 01605, USA.; ^3^ Department of Neurosurgery, Department of Physiology and Pharmacology, Department of Neurosurgery and Anesthesiology, School of Medicine, Loma Linda University, Risley Hall, Room 219, 11041 Campus Street, Loma Linda, CA 92354, USA.; ^4^ Fujian Key Laboratory of Toxicant and Drug Toxicology, Medical College, Ningde Normal University, Ningde, China.; ^5^ Science and Technology Innovation Center, Shandong First Medical University & Shandong Academy of Medical Science, Jinan, China.; ^6^ School of Traditional Chinese Medicine, Shandong First Medical University & Shandong Academy of Medical Science, Jinan, China.; ^7^ Neck-Shoulder and Lumbocrural Pain Hospital of Shandong First Medical University, Shandong First Medical University & Shandong Academy of Medical Science, Jinan, China.; ^8^ State Key Laboratory of Systems Medicine for Cancer, Shanghai Cancer Institute, Renji Hospital, Shanghai Jiao Tong University School of Medicine, Shanghai 200127, China.; ^9^ Beijing University of Chinese Medicine, Beijing, China.; ^10^ Ningde Normal University; Department of Vascular and Interventional Radiology, Ningde Municipal Hospital of Ningde Normal University; Fujian Key Laboratory of Toxicant and Drug Toxicology, Medical College, Ningde Normal University; Fujian Key Laboratory of Medical Bioinformatics, Fujian Medical University, Fuzhou, China.

## Abstract

Osteoporosis is a systemic skeletal disorder characterized by reduced bone mass, impaired microarchitecture, and increased fracture risk, primarily resulting from dysregulated bone remodeling. Increasing evidence highlights a close interaction between bone metabolism and the gut microbiota. Alterations in bone mineral density can influence gut microbial composition. Conversely, microbial dysbiosis disrupts bone homeostasis through multiple pathways, including microbial metabolites, immune regulation, and neuroendocrine signaling. Short-chain fatty acids suppress osteoclast differentiation and enhance intestinal calcium absorption, while gut dysbiosis promotes bone loss by impairing intestinal barrier integrity and increasing proinflammatory cytokines such as tumor necrosis factor-α and interleukin-6. The gut–brain–bone axis represents an important regulatory network linking the central nervous system, gut-derived signals, and skeletal remodeling. Chronic stress and neurodegenerative conditions activate the hypothalamic–pituitary–adrenal axis and bone-derived extracellular vesicle signaling, thereby favoring bone resorption. Estrogen deficiency further disrupts the receptor activator of nuclear factor κΒ ligand/osteoprotegerin signaling pathway and alters gut microbial composition, contributing to postmenopausal bone loss. Therapeutic strategies targeting this axis, including probiotics, prebiotics, fecal microbiota transplantation, dietary fiber supplementation, and pharmacological or natural compounds, show potential in restoring microbial balance and improving bone metabolism. Future studies integrating multiomics approaches and well-designed clinical trials are needed to clarify microbiome–bone interactions and support the development of targeted interventions for osteoporosis.

## Introduction

**Fig. 1.**
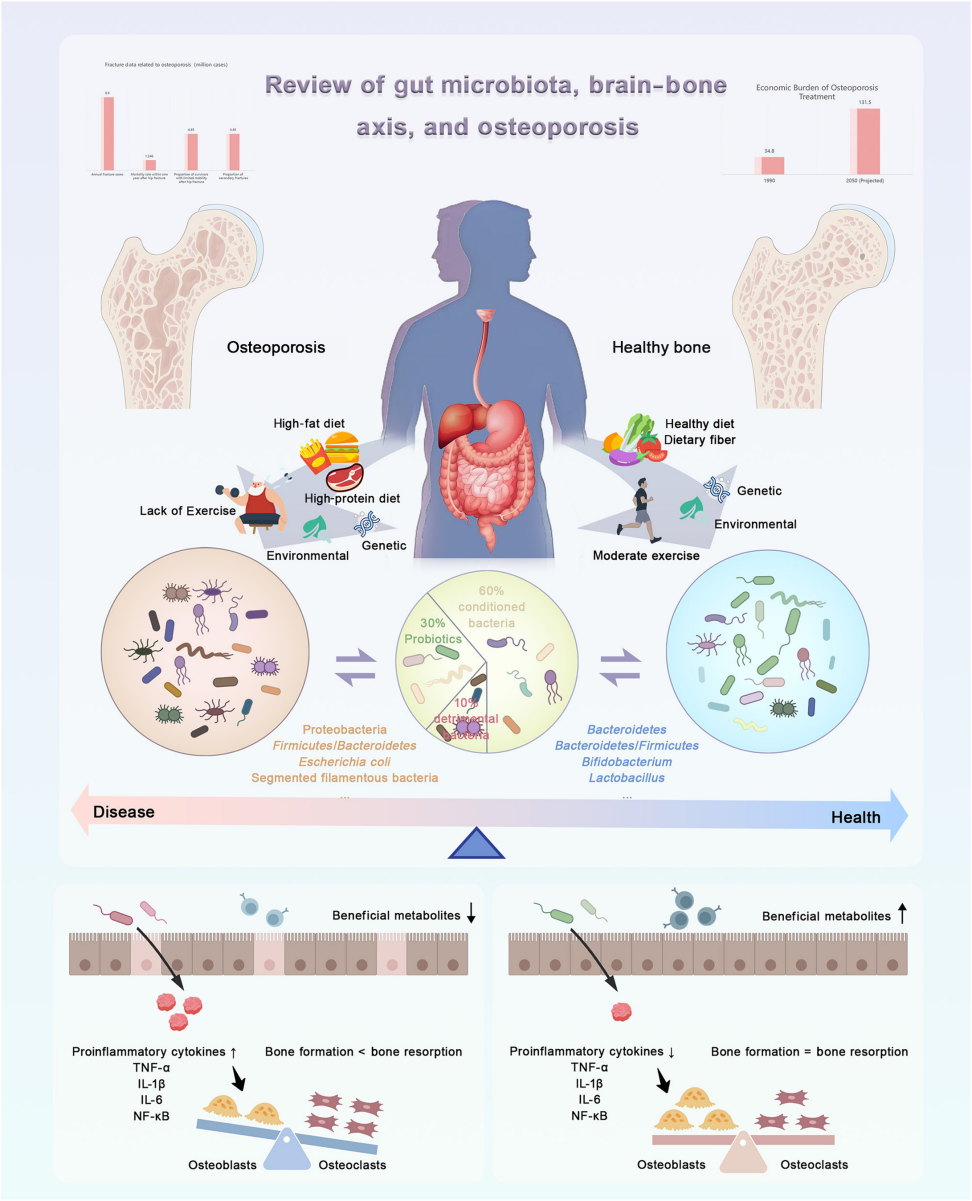
Gut microbiota–brain–bone axis in OP. Pathological factors disrupt gut microbial homeostasis, promote inflammation, and accelerate bone loss, whereas moderate exercise, healthy diet, and probiotics restore beneficial microbiota, suppress inflammation, and support bone remodeling through gut–bone bidirectional regulation.

Osteoporosis (OP) is a systemic skeletal disorder characterized by reduced bone mass and impaired microarchitecture, leading to increased fracture risk [1]. Bone integrity is maintained through a tightly regulated balance between bone formation and resorption. Estrogen deficiency during menopause, along with genetic, age, sex, and familial factors, disrupts bone remodeling, increasing osteoclast activity, reducing osteogenic regulation, and leading to bone fragility and broader clinical impacts. Fractures of the vertebrae, hip, and wrist are often associated with persistent pain, functional impairment, and a marked decline in quality of life [2]. Once bone homeostasis is disrupted, the likelihood of subsequent fractures also rises [3]. From a public health perspective, OP imposes a substantial burden on healthcare systems by driving higher healthcare utilization, long-term therapeutic needs, and productivity losses, a challenge that is expected to escalate as global populations continue to age [4]. Although pharmacological interventions and nutritional supplementation are effective in slowing bone loss, their prolonged use may be constrained by side effects, underscoring the need for safer and more sustainable preventive strategies. As a result, growing attention has shifted toward nonpharmacological and system-oriented approaches. In particular, physical activity has been recognized as an important regulator of bone strength and inflammatory status, reflecting the close interplay among metabolic, immune, and skeletal systems [5,6]. At the same time, advances in epigenetics and metabolic research have highlighted their roles in shaping immune responses within the bone microenvironment, suggesting that OP arises from coordinated dysregulation across multiple pathways rather than from a single pathogenic factor [7]. Consequently, prevention and early intervention, particularly in high-risk populations, have become key priorities in contemporary OP research (Fig. 1).

Within this broader framework, the gut microbiota has emerged as a critical mediator linking metabolic processes, immune regulation, and distant organ systems. As a host-associated microbial ecosystem, the gut microbiota contributes to metabolic balance and immune homeostasis while communicating with extraintestinal tissues through neuroendocrine pathways, including the gut–brain and gut–muscle axes [8]. Its composition is influenced not only by host genetics but also by modifiable factors such as diet, physical activity, and environmental exposures (Fig. 1) [9]. Accumulating evidence has associated alterations in microbial composition and function with OP, supporting microbiota modulation as a complementary strategy for disease prevention and treatment. Mechanistically, microbiota-derived metabolites, particularly short-chain fatty acids (SCFAs), interact with immune and endocrine signaling to regulate osteoblast and osteoclast activity, inflammatory tone, and mineral utilization. These interactions influence bone remodeling in a manner that reflects host metabolic and immune status. Accordingly, interventions targeting the gut microbiota, including probiotics, prebiotics, synbiotics, and fecal microbiota transplantation (FMT), have been explored for their capacity to restore microbial balance and attenuate bone loss. Although most evidence to date comes from experimental models, the translational potential of these approaches is increasingly acknowledged [10,11]. Beyond local gut–bone interactions, emerging research has proposed the existence of a gut–brain–bone axis that integrates neural, immune, and endocrine signaling (Fig. 1) [12,13]. Within this framework, stress-related neural inputs affect gut function, whereas gut-derived metabolites and immune mediators modulate central neuroendocrine pathways involved in skeletal remodeling.

At the host level, OP reflects the combined effects of endocrine imbalance, immune dysregulation, nutritional factors, and environmental influences. Aging, menopause, hormonal disorders, and long-term exposure to certain medications contribute to bone loss by reducing osteoblast activity and enhancing bone resorption. Increasing evidence suggests that these risk factors are accompanied by changes in gut microbial metabolism, including altered production of SCFAs, bile acids, and vitamins. Such changes may further promote systemic inflammation and impair bone mineralization [14]. OP is increasingly recognized as a multifactorial disorder that is shaped by overlapping microbial, endocrine, and immune pathways that together define disease trajectory and therapeutic opportunity [15]. This review synthesizes current evidence supporting the gut–brain–bone axis as an integrative framework for understanding OP. Rather than viewing bone loss as an isolated skeletal event, we emphasize the coordinated interactions between gut microbiota, immune signaling, and central neuroendocrine regulation. We examine how dysbiosis, intestinal barrier dysfunction, and altered microbial metabolism reshape osteoimmune communication and bone remodeling, as well as how neurobiological pathways such as vagal signaling, neuroinflammation, the stress-responsive hypothalamic–pituitary–adrenal (HPA) axis contribute to the clinical relevance of this framework. Finally, we outline future directions that prioritize multiomics integration, multikingdom microbiome profiling, and system-level and artificial-intelligence-assisted precision strategies, with the goal of enabling individualized, microbiome-informed prevention and management of OP.

## Gut Microbiota and OP

OP is a systemic skeletal disorder characterized by reduced bone mass and microarchitectural deterioration, leading to increased fracture susceptibility. Growing evidence indicates that the gut microbiota acts as a system-level regulator of bone homeostasis, positioning the gut–bone axis as a key pathway in OP. Microbial community structure and metabolic output are shaped by diet, aging, and lifestyle exposures and, in turn, influence host endocrine and immune programs that govern bone remodeling. When dysbiosis emerges with aging, barrier integrity becomes compromised, systemic inflammatory tone rises, and the profile of microbial metabolites, including SCFAs, shifts. These coordinated perturbations impair osteoblast function while promoting osteoclast activity, thereby mechanistically linking gut microbial ecology to skeletal fragility. Accordingly, the gut microbiota is increasingly viewed as both a susceptibility biomarker and a therapeutic target in OP, and microbiota-directed strategies such as probiotics, prebiotics, and dietary or lifestyle modulation show potential to restore bone-relevant signaling in both experimental and clinical contexts.

### Gut microbiota and host health: Integrated regulation of metabolism, immunity, and bone

**Fig. 2.**
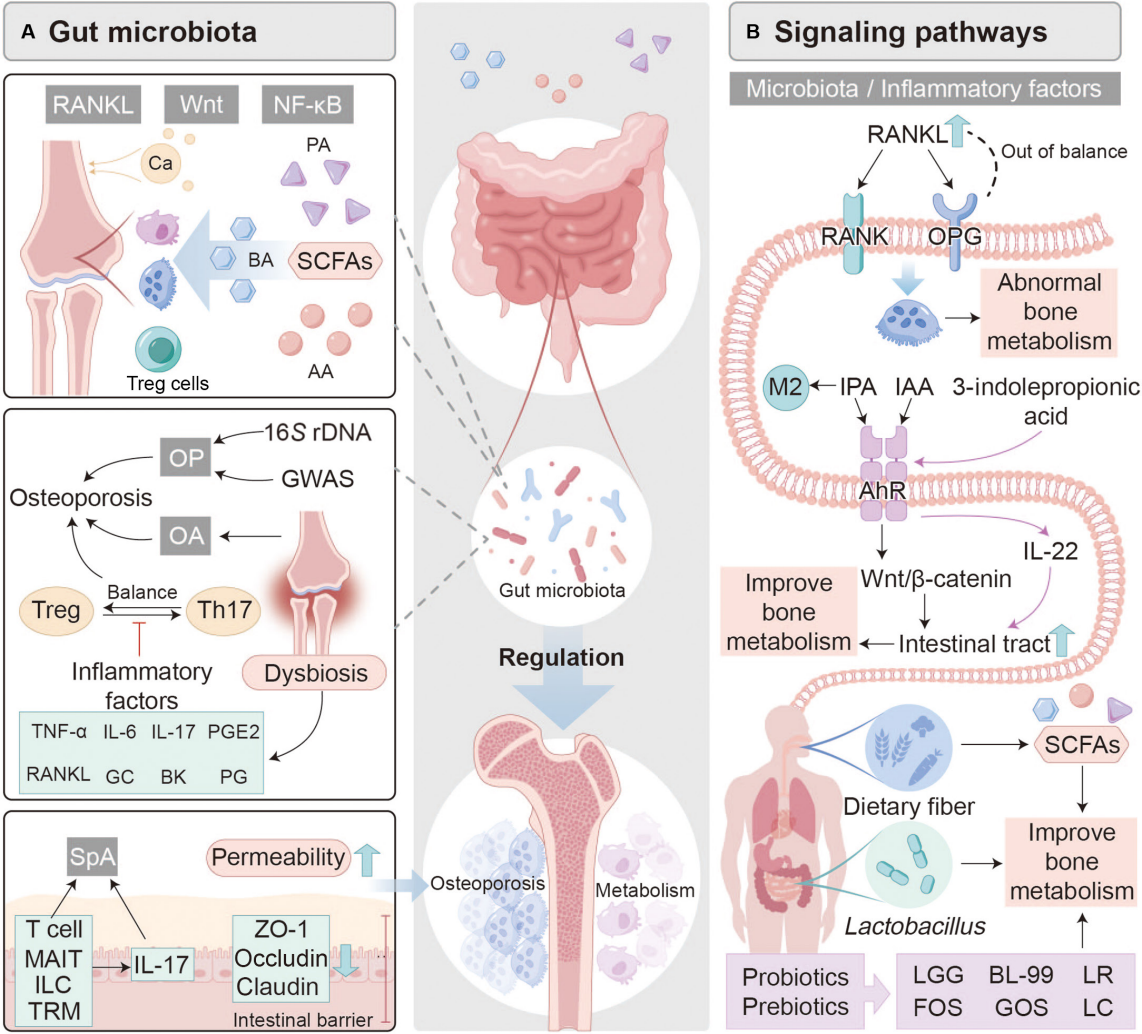
Gut microbiota–immune–bone signaling in OP and metabolic bone disorders. (A) Microbial metabolites, including SCFAs, bile acids, and amino acids, maintain immune homeostasis by modulating the Treg/Th17 cell balance. Gut dysbiosis identified by 16*S* rRNA gene sequencing and genome-wide association studies (GWASs) disrupts this balance, elevating TNF-α and IL-6, enhancing RANKL expression, and promoting bone resorption. Increased intestinal permeability further amplifies systemic inflammation in inflammatory bone disorders. (B) Microbiota-derived indoles activate key bone regulatory pathways, including RANKL–RANK–OPG and Wnt/β-catenin signaling. Probiotics and prebiotics strengthen intestinal barrier integrity, suppress inflammatory cytokines, and support bone formation, highlighting microbiota modulation as a potential therapeutic strategy for OP. PA, propionic acid; BA, bile acids; AA, arachidonic acid; PGE2, prostaglandin E2; GC, glucocorticoids; BK, bradykinin; PG, prostaglandins; SpA, short-chain fatty acid–producing bacteria; MAIT, mucosal-associated invariant T cells; ILC, innate lymphoid cells; TRM, tissue-resident memory T cells; ZO-1, zonula occludens-1; LGG, *Lactobacillus rhamnosus* GG; BL-99, *Bifidobacterium lactis* BL-99; LR, *Lactobacillus rhamnosus*; LC, *Lactobacillus casei*; GOS, galactooligosaccharides; FOS, fructooligosaccharides.

The gut microbiota constitutes a complex symbiotic ecosystem that is essential for maintaining intestinal barrier integrity, immune tolerance, and metabolic homeostasis. Under physiological conditions, microbial communities regulate epithelial permeability, limit excessive inflammatory responses, and facilitate nutrient metabolism. In contrast, dysbiosis is associated with metabolic imbalance and a broad spectrum of chronic disorders (Fig. 2). These systemic consequences extend to the skeletal system, where bone metabolism is increasingly recognized as a downstream target of microbial regulation. Microbiota-derived metabolites can influence osteoblast and osteoclast function directly, while dysbiosis may impair mineral utilization, alter growth factor signaling, and perturb osteoimmune pathways such as the osteoprotegerin (OPG) and receptor activator of nuclear factor κΒ ligand (RANKL) axis, which collectively promote bone loss [10]. In parallel, gut microbial imbalance may compromise neuromuscular function and skeletal muscle maintenance, thereby indirectly weakening bone quality [16].

Immune regulation represents a central mechanism through which the gut microbiota influences skeletal outcomes. In homeostasis, commensals calibrate innate and adaptive immunity; in dysbiosis, chronic inflammatory programs are amplified, proinflammatory cytokine production increases, and bone resorption is facilitated. Experimental models further suggest that systemic exposure to microbial products can trigger peripheral immune activation and reprogram bone marrow T cell populations, contributing to inflammatory bone injury [17]. Beyond bacteria, the gut mycobiome and virome also regulate bone metabolism. In estrogen deficiency and metabolic disorders, fungal expansion and phage remodeling exacerbate bacterial dysbiosis, immune imbalance, and altered metabolite availability [18]. Fungal components and bacteriophage-driven bacterial shifts influence the balance between osteoclast and osteoblast activity through immune pathways and SCFA metabolism [19]. These findings indicate that skeletal homeostasis is regulated by a coordinated, multikingdom gut ecosystem rather than bacteria alone.

### Microbiota-derived metabolites and bone remodeling

Gut-microbiota-derived metabolites constitute a critical biochemical interface linking intestinal ecology to postnatal skeletal development and lifelong bone remodeling. Dysbiosis can drive pathological bone loss, and modulation of the microbiota through probiotics or prebiotics has been proposed as a route to reverse maladaptive signaling [20]. SCFAs, produced through fermentation of dietary fibers, serve as key metabolic mediators that nourish the intestinal barrier while influencing mineral utilization, immune balance, and inflammatory tone (Table 1 and Fig. 2). Among these metabolites, butyrate has been linked to regulatory T (Treg) cell differentiation, suppression of osteoclastogenesis, and support of osteoblast activity, thereby stabilizing bone remodeling [21]. SCFAs may stimulate anabolic endocrine signals, including pathways involving insulin-like growth factor and glucagon-like peptide signaling, and can engage cell surface receptors that converge on osteogenic and antiresorptive programs such as Wnt-dependent pathways. Trimethylamine *N*-oxide (TMAO) has been associated with OP and may contribute to inflammatory signaling and impaired bone formation via nuclear factor κB (NF-κB) signaling mechanisms [22]. Moreover, microbiota-derived bile acid metabolites have been implicated in bone remodeling by modulating osteoclast differentiation and inflammatory responses, indicating that the influence of microbial metabolites extends beyond SCFAs. Emerging evidence further suggests that targeted regulation of microbial metabolic activity may mitigate systemic inflammation associated with aging, with potential implications for skeletal health [23].

**Table 1.** Intestinal flora regulates bone metabolism in multiple dimensions

Key metabolites	Example	Mechanism of action	Effects
**Bacterial metabolites**			
SCFAs	Acetic acid, propionic acid, butyric acid	Regulate calcium absorption, immune balance, and inflammatory response	Promote bone metabolism
		Lower intestinal pH, enhance the expression of calcium-binding proteins, and increase mineral solubility	Improve calcium absorption efficiency
		Regulate the differentiation of Treg cells	Reduce osteoclastogenesis and stimulate osteoblast activity
		Activate IGF-1 and GLP-1 secretion	Indirectly promote bone formation
		Bind to G-protein-coupled receptors on the cell surface	Inhibit osteoclast differentiation
		Enhance osteogenesis via the Wnt signaling pathway	Enhance osteoblast activity
TMAO	–	Activate the NF-κB signaling pathway	Promote inflammation and inhibit bone formation
**Gut microbiota imbalance**			
–	–	Modulates immune responses, metabolites, and cellular processes	Regulates the occurrence of OP
		Reduces the expression of inflammatory factors (such as TNF-α and IL-6) and matrix metalloproteinase-13	
		Regulates bone mineral density (BMD)	
**Intestinal barrier function**		
–	–	Intestinal barrier integrity regulates immune system responses	Directly affects the balance of bone metabolism
		Damage to the intestinal barrier increases inflammatory responses and affects immune cell activation	Disrupts the balance between bone resorption and formation, promoting the occurrence of OP
**Regulation of inflammatory factors**			
Proinflammatory cytokines	TNF-α, IL-6, IL-17, RANKL	Trigger an immune response	Activate osteoclasts, promoting bone loss
		Activate the NF-κB signaling pathway	Regulate bone resorption and promote the occurrence of OP
Inflammatory mediators	Glucocorticoids, bradykinin, prostaglandins	Regulate RANKL expression	Further activate osteoclasts

### Dysbiosis in OP

Recent investigations support dysbiosis as a meaningful contributor to the initiation and progression of OP [24]. Human research has revealed distinct gut microbiota features in individuals with OP compared to healthy controls, with these microbial shifts correlating with variations in bone mineral density and metabolite profiles at clinically relevant skeletal sites, thereby supporting the involvement of immune and metabolic pathways and suggesting their potential as biomarkers [25]. Collectively, these findings indicate that OP is influenced not only by host-intrinsic factors but also by the structure and function of the gut ecosystem, revealing potential targets for therapeutic intervention.

### Gut barrier function and bone resorption

The intestinal barrier is a multilayered defense system that controls microbial containment, nutrient absorption, and immune exposure. Disruption of this barrier is linked to inflammatory bowel disease and systemic disorders, including OP, by increasing intestinal permeability, systemic inflammation, and bone loss. Under conditions such as spondyloarthritis and bowel inflammation, barrier dysfunction and tight junction dysregulation promote microbial translocation and immune activation. Animal studies suggest that restoring microbial homeostasis may enhance barrier integrity, reduce inflammation, and mitigate bone loss [26]. Recent findings also highlight specific microbial metabolites, including branched SCFAs such as isobutyrate, as potential contributors to barrier support and inflammation regulation in intestinal disease models [27]. Therapeutic strategies that aim to reshape gut microbiota composition while restoring barrier integrity may offer a promising approach to attenuating inflammation-driven bone resorption in OP.

### The role of inflammatory factors in gut microbiota dysbiosis-induced OP

Gut microbial dysbiosis has been increasingly implicated in the pathogenesis of OP through immune-mediated mechanisms that shift bone remodeling toward net bone loss. Disruption of intestinal barrier integrity facilitates the translocation of microbial products into systemic circulation, thereby enhancing systemic inflammatory tone [28]. In this setting, proinflammatory cytokines such as tumor necrosis factor-α (TNF-α) and interleukin-6 (IL-6) promote osteoclast differentiation and activation while suppressing osteoblast function, ultimately accelerating bone resorption [29]. Moreover, inflammatory signaling can alter bone marrow immune programs, particularly by modulating the balance of T helper subsets and Treg cells, which, in turn, influences osteoclastogenesis through cytokine- and RANKL-dependent pathways. As mentioned earlier, microbiota-derived metabolites, such as SCFAs, may counteract these processes by promoting anti-inflammatory immune regulation. Dysbiosis-driven inflammation acts as a key mediator linking gut microbial disruption to skeletal fragility, highlighting cytokine pathways as potential targets for OP intervention.

### Regulation of osteoclast activity and bone metabolism by the RANK/RANKL/OPG system

Skeletal homeostasis depends on tightly coordinated signaling networks that regulate osteoblast–osteoclast coupling, most notably the Wnt/β-catenin pathway and the RANK/RANKL/OPG axis (Fig. 2). Emerging evidence suggests that gut microbiota can influence bone metabolism by perturbing Wnt signaling and enhancing osteoclast activity [30]. Within the RANK/RANKL/OPG system, RANKL drives osteoclastogenesis by engaging RANK on osteoclast precursors, whereas OPG acts as a decoy receptor that inhibits this interaction and limits bone resorption. An increased ratio of bioavailable RANKL to OPG favors osteoclast activation and contributes to the development of OP. Inflammatory cytokines, including TNF-α, IL-6, and IL-17, can further amplify this imbalance by reinforcing resorptive programs and by reshaping the gut microbial milieu, creating a feedforward loop between dysbiosis and bone loss.

Microbiota-oriented strategies have been explored to interrupt this loop. Preclinical studies suggest that gold nanoparticles may modulate gut microbial composition and related metabolites, including TMAO, leading to reduced proinflammatory cytokine production and attenuation of osteoclastogenesis via a putative microbiota–TMAO–immune axis [31]. However, clinical evidence remains insufficient, and safety and efficacy in human OP require careful evaluation. Beyond metabolites, specific microbes may influence endocrine-immune cross-talk; for example, segmented filamentous bacteria can affect parathyroid hormone signaling, alter intestinal immune programs, and promote immune cell trafficking to the bone marrow where RANKL-mediated resorption is enhanced. Moreover, increased intestinal permeability can facilitate the translocation of lipopolysaccharide into the systemic circulation, sustaining chronic inflammation and favoring osteoclast-driven bone loss [32]. These findings support the concept that gut microbiota regulate bone remodeling not only through the RANKL/RANK/OPG axis but also via integrated immune and neuroendocrine pathways, potentially involving the gut–brain axis.

### Aryl hydrocarbon receptor-mediated tryptophan metabolism in gut barrier integrity and bone remodeling

Tryptophan-derived metabolites, which serve as endogenous ligands for the aryl hydrocarbon receptor (AhR), regulate microbial homeostasis in the gut and influence bone metabolism through microbiota-dependent mechanisms (Fig. 2) [33]. Microbial metabolites, such as SCFAs and tryptophan metabolites, can enhance gut barrier function. Indole-3-acetic acid (IAA) and indole-3-propionic acid (IPA) have been shown to improve gut barrier integrity via AhR activation and thereby promote bone health. IAA activates AhR signaling in intestinal epithelial cells, leading to stimulation of the Wnt/β-catenin pathway, which promotes epithelial regeneration and accelerates repair of the intestinal barrier [34]. Concurrently, indole metabolites may promote anti-inflammatory immune polarization, characterized by M2 macrophage differentiation and increased IL-10 production, processes that support osteoblast differentiation and suppress osteoclastogenesis [35]. Beyond the indole pathway, the kynurenine pathway also links tryptophan metabolism to immune regulation and bone remodeling. Dysregulated kynurenine metabolism has been associated with inflammatory and metabolic states relevant to OP, and microbiota-bile acid interactions may further modulate intestinal farnesoid X receptor signaling and inflammatory tone [36]. Functionally, certain kynurenine metabolites are associated with oxidative stress and resorptive bias, whereas indole-related metabolites such as IPA and kynurenic acid (KYNA) may suppress NF-κB activation and reduce proinflammatory cytokine expression, helping preserve bone remodeling balance [37]. Collectively, these findings support microbiota-informed therapeutic strategies targeting tryptophan–AhR metabolism to strengthen gut barrier function, restore immune homeostasis, and stabilize bone turnover.

### The link between high-fiber diets and SCFA production

Dietary fiber promotes microbial fermentation and supports SCFA generation, which contributes to barrier integrity, immune balance, and bone remodeling. SCFAs, such as acetate, propionate, and butyrate, can suppress osteoclast activity and support osteoblast function through immunometabolic regulation. Milk-derived extracellular vesicles have been reported to shape gut microbial composition in experimental OP models, enriching beneficial taxa and increasing SCFA availability [38]. Higher SCFA levels may strengthen barrier function, reduce endotoxin leakage, and shift cytokine profiles toward an anti-inflammatory state, thereby limiting osteoclastogenesis and mitigating bone loss [39]. Dietary fiber may similarly support these effects by remodeling community structure and promoting SCFA-related functions [40]. SCFAs can also signal through receptors on intestinal and immune cells, including G-protein-coupled receptor 43 and G-protein-coupled receptor 41, coordinating metabolic, immune, and endocrine cues that influence cytokine output and bone remodeling [41]. Together, these findings support fiber-based dietary strategies as a viable approach to modulate the gut–bone axis in OP prevention.

### The prospects of specific strains for OP intervention

Probiotic interventions for OP are increasingly viewed as strain-specific rather than genus-level approaches. Experimental evidence suggests that *Lactobacillus rhamnosus* GG can improve bone-related outcomes in estrogen-deficient models, consistent with immunomodulatory effects that reduce inflammatory cytokines and rebalance T helper 17 (Th17) and Treg cell programs [42]. In contrast, *Bifidobacterium* species may exert a stronger influence through metabolic functions, including the enhancement of SCFA production and barrier support. These mechanistic differences argue for functional classification and strain-resolved evaluation when selecting probiotics for OP. Mechanistically, candidate strains may affect markers of bone turnover, improve junctional integrity, and partially reverse dysbiosis associated with estrogen deficiency, suggesting that microbiota remodeling plays a crucial role in skeletal health. Potential improvements in bone-related measures with probiotic supplementation may occur, but responses may vary with baseline microbiota, host context, and intervention design. Probiotics may support OP management by restoring barrier function, reducing inflammation, and shifting osteoimmune signaling toward remodeling balance, yet their use requires careful strain selection and responder stratification [43].

## Brain–Bone Axis and OP Pathogenesis

### Gut hormones in bone metabolism

The gastrointestinal tract secretes multiple hormones, including glucagon-like peptide-1 (GLP-1), GLP-2, glucose-dependent insulinotropic peptide (GIP), and peptide YY, all of which play important roles in bone metabolism. These hormones regulate skeletal remodeling by binding to their respective receptors, namely, the GLP-1 receptor, GLP-2 receptor, GIP receptor, and Y1 receptor, thereby influencing osteoblast and osteoclast activity [44]. Accumulating evidence indicates that GLP-1, GLP-2, and GIP suppress bone resorption, while GIP additionally promotes bone formation. Importantly, growing evidence suggests that the gut microbiota interacts closely with these hormonal pathways, thereby modulating bone metabolism through coordinated effects on nutrient absorption, immune regulation, and endocrine signaling cascades.

GLP-1, for example, has been shown to protect skeletal integrity under diabetic conditions by improving insulin sensitivity and regulating gastric emptying. Clinical and experimental studies using GLP-1 receptor agonists, such as liraglutide, demonstrate reduced markers of bone resorption and a lower incidence of fractures in patients with OP [45]. These findings suggest that GLP-1-based therapies may help prevent OP in diabetic populations by enhancing osteoblast activity and inhibiting osteoclast differentiation. Similarly, GIP contributes to skeletal development and the improvement of bone density and has shown beneficial effects in postmenopausal women and patients with short-bowel syndrome. Combined GLP-1 and GIP therapies therefore represent a promising strategy for addressing the co-occurrence of diabetes and OP by simultaneously improving glucose homeostasis and skeletal health. GLP-2 also plays a critical role in bone metabolism by maintaining markers of bone formation and inhibiting bone resorption, particularly in patients with intestinal malabsorption syndromes. Because of its capacity to enhance trabecular microarchitecture and prevent bone loss, GLP-2 has potential therapeutic relevance in postmenopausal OP (PMOP). At the molecular level, gastrointestinal hormones exert their effects on the bone via signaling pathways such as Wnt/β-catenin, phosphoinositide 3-kinase, and mitogen-activated protein kinase, which promote osteoblast proliferation and inhibit osteoclastogenesis. Overall, gut hormones function as anabolic regulators of bone metabolism, and their interaction with the gut microbiota provides new opportunities for microbiota-informed prevention and treatment strategies in OP.

### Neuroendocrine disruptions and estrogen deficiency in OP

A primary factor contributing to the development of OP is the decline in estrogen levels after menopause. Estrogen impacts bone metabolism through multiple mechanisms, including immune modulation and the regulation of osteoblast and osteoclast function. Chronic estrogen deficiency disrupts these regulatory mechanisms, leading to increased osteoclast differentiation and accelerated bone loss. Specifically, estrogen deficiency activates the RANKL/RANK/OPG signaling pathway, thereby promoting osteoclastogenesis and increasing bone resorption (Table 2) [46]. In addition, loss of estrogen can alter the intestinal mucosa, increasing gut permeability and allowing toxic metabolites such as lipopolysaccharides to be released into circulation. This process triggers inflammatory responses that further stimulate osteoclast formation. In ovariectomized animal models, the depletion of estrogen markedly disturbs the gut microbiome, with alterations closely associated with bone loss. *Bacteroides*, *Pseudomonas aeruginosa*, and *Faecalibacterium prausnitzii* have been associated with bone loss, whereas *Lactobacillus* and *Butyrivibrio* species have been linked to protective effects against bone resorption [47].

**Table 2.** The brain–bone axis regulates bone metabolism

Source and function	Mechanism	Effects
**5-HT**		
Intestinal	Modulation of intestinal microbiota affects the expression of TPH1	Inhibits the activity of peripheral bone cells, thereby inhibiting bone formation
Brain	Regulates hypothalamic neurons through 5-HT2C receptors, further regulating sympathetic nerve activity	Promotes osteoblast proliferation and maintenance of bone density
**Neuropeptide Y**		
Osteocytes	Regulates the differentiation of mesenchymal stem cells and promotes the formation of adipocytes	Causes bone loss
**Peptide YY**		
Intestinal	Inhibits bone formation	Regulates bone density
**GLP-1**		
Intestinal L cells	Activates the cyclic adenosine monophosphate (cAMP) and AKT pathways	Promotes the expression of osteogenic genes and increases bone formation
	Inhibits osteoclast formation	Reduces bone resorption
**GLP-2**		
Intestinal	Inhibits the inflammatory response and improves trabecular bone microstructure	Increases bone density and reduces bone resorption
**GIP**		
Intestinal	Inhibits bone resorption	Reduces bone loss and enhances bone strength
**Glutamate**		
Brain, gut microbes	Interacts with *N*-methyl-D-aspartate receptors	Regulates bone remodeling
Calcitonin gene-related peptide		
Nervous system	Activates cAMP or protein kinase C pathways and up-regulates IGF-1 in osteoblasts	Stimulates bone formation and activates bone marrow stem cell division
**Cortisol**		
Adrenal cortex	Regulates immune cell activity and cytokine release	Promotes bone resorption and inhibits bone formation, leading to OP
	Affects intestinal barrier function, leading to increased intestinal permeability	
	Directly affects the function of bone cells (such as osteoclasts and osteoblasts)	
**Estrogen**		
Ovaries	Regulates Wnt/β-catenin and Notch signaling pathways	Promotes the differentiation of bone marrow mesenchymal stem cells into osteoblasts
	In the absence of ovarian estrogen, the RANKL/RANK/OPG signaling pathway is activated	Promotes the formation and activity of osteoclasts
	Causes an intestinal microbiome imbalance	Increases the presence of bacteria associated with bone loss (such as *Oncoccus* and *Clostridium*)
	Estrogen deficiency damages the intestinal barrier	Increases intestinal permeability, triggers a systemic inflammatory response, and enhances bone resorption

Estrogen also maintains immune homeostasis by regulating T cell differentiation. It promotes the activation of Treg cells, which inhibit osteoclastogenesis, while suppressing Th17 cell differentiation to prevent excessive bone resorption. In the absence of estrogen, the balance between Treg and Th17 cells is disrupted, resulting in elevated levels of proinflammatory cytokines, including TNF-α and IL-17. These cytokines accelerate osteoclast activity and bone degradation. Emerging evidence suggests that estrogen-deficiency-induced alterations in the gut microbiota further amplify the production of osteoclastogenic mediators, such as RANKL and TNF-α, thereby exacerbating bone loss. Recent findings indicate that the gut–brain axis is a novel regulator of OP pathophysiology. A decrease in estrogen may change the signaling through the hypothalamic–pituitary axis and may affect systemic immune function and bone metabolism [48]. Estrogen deficiency has been shown to increase Th17 cell populations in the bone marrow, ultimately promoting osteoclastogenesis and bone resorption. Collectively, estrogen deficiency contributes to OP by disrupting immune regulation, altering the gut microbiota, and impairing neuroendocrine homeostasis. The gut microbiota plays a central role in the integration of these three physiological systems. Elucidating these interactions may facilitate the development of microbiota-targeted interventions for restoring bone health and preventing OP.

## The Interaction Between the Gut Microbiota and the Brain–Gut Axis

### Microbial metabolites reaching the brain and shaping central regulation

Microbiota-derived metabolites, particularly SCFAs such as butyrate, exert regulatory effects on both skeletal homeostasis and central nervous system function. Beyond local gut effects, these metabolites may reach the brain through barrier transport, vagal signaling, and immune-mediated routes [49], thereby integrating metabolic cues with neuroregulatory programs relevant to OP. Upon reaching central regulatory centers, these microbial-derived signals can influence hypothalamic integration, autonomic tone, and neuroendocrine set points that ultimately shape skeletal remodeling. From a therapeutic perspective, this capacity positions microbial metabolites as modulators that can simultaneously target gut barrier integrity, immune tone, and central regulation, rather than acting solely on bone-local pathways. Environmental stressors can disturb microbial ecology and reduce butyrate-producing capacity, creating a context in which metabolite availability, barrier integrity, and immune tone shift in the same direction. Observational evidence has linked higher dietary butyrate equivalents to reduced risk of OP under conditions of elevated toxic exposure, while experimental work suggests that butyrate supplementation can improve trabecular architecture, strengthen gut barrier function, and increase Treg cell proportions in exposed models. These findings collectively suggest that restoring metabolite-mediated gut–brain communication may represent a preventive strategy for OP in populations exposed to chronic environmental or metabolic stressors. Overall, the evidence supports the concept that microbial-derived molecules function as systemic messengers linking the gut, muscle, bone, and brain through coordinated immunometabolic and neuroregulatory pathways [50]. Consistent with this view, neonatal neurotoxicity models have shown that restoring the microbiota–metabolite–brain connection through FMT and bile acid supplementation can reverse social and synaptic impairments, underscoring the capacity of microbial metabolites to shape neural function and behavior [51]. Although derived from nonosteoporotic models, these data highlight the plasticity of the gut–brain interface and support its relevance as an upstream regulatory node with implications for long-term skeletal health.

### Microbiota, neurotransmitters, and bone remodeling

The gut–brain axis is a bidirectional communication network linking the gastrointestinal tract and the central nervous system through neural, endocrine, immune, and metabolic pathways. A defining feature of brain–bone coupling within the gut–brain axis is the functional divergence between peripheral and central neurotransmitter actions on skeletal homeostasis. In the context of bone homeostasis, this axis is notable because microbial signals can influence both central- and peripheral-regulatory neuroendocrine mechanisms that shape osteoblast and osteoclast activity. Importantly, these mechanisms operate through distinct anatomical levels and temporal dynamics during OP progression and treatment, expanding the conceptual framework of OP from a bone-centered disorder to a system-level disease influenced by multilevel neuroendocrine regulation. Microbial metabolites, including SCFAs, contribute to bone remodeling through immune and metabolic routes as reviewed above, but in this section, the emphasis is on neuroactive communication pathways that differentially engage peripheral sensors and central integrative circuits, including blood–brain-barrier-related signaling, vagal pathways, and neurotransmitter regulation [52]. Serotonin provides a clear example of peripheral–central duality within the gut–brain–bone axis. At the peripheral level, serotonin produced by enterochromaffin cells inhibits osteoblast proliferation and bone formation through endocrine actions on skeletal targets. In contrast, at the central level, serotonergic circuits within the brain can promote bone formation by reducing sympathetic outflow to the skeleton, thereby favoring osteoblast activity and limiting bone resorption. Gut microbes and their metabolites can modulate serotonin biology by influencing tryptophan availability, enterochromaffin cell activity, and the expression of serotonin biosynthetic enzymes such as tryptophan hydroxylase 1 (TPH1), thereby altering peripheral serotonin tone [53]. In parallel, microbiota-derived signals may indirectly influence central serotonergic signaling through vagal pathways and immune-to-brain communication, highlighting that dysbiosis can shift the balance between bone-inhibitory peripheral signals and bone-protective central regulatory circuits. Beyond neurotransmitter regulation, dysbiosis can also increase systemic exposure to microbial ligands that activate innate immune sensing pathways, which may indirectly perturb neuroendocrine regulation and promote osteoclastogenic signaling. At the peripheral level, this manifests as enhanced inflammatory cues that favor osteoclast differentiation and activity. At the central level, sustained immune activation may engage neuroinflammatory pathways that further amplify skeletal catabolism. Neuroinflammation represents an additional node linking gut dysbiosis to skeletal loss. Under conditions of central immune activation, dysbiosis may enhance inflammatory inputs to the brain through humoral routes and vagal afferents, amplifying microglial and astrocytic activation. Activated glial cells increase proinflammatory mediators, including TNF-α, IL-1β, and IL-6, which can favor osteoclast differentiation and activity by reinforcing osteoclastogenic signaling and can impair osteoblast maturation by suppressing osteogenic pathways. These central inflammatory mechanisms may be especially relevant to OP progression, whereas peripheral immune and neurotransmitter pathways may represent more accessible targets during therapeutic intervention. Collectively, these observations suggest that interventions targeting neurotransmitter balance and neuroinflammatory tone through microbiota modulation may complement conventional antiresorptive or anabolic therapies. Such strategies may act predominantly through peripheral regulatory pathways, while secondarily normalizing maladaptive central neuroendocrine circuits, particularly in patients with comorbid stress or neuropsychiatric conditions. Together, these observations support a gut–brain–bone framework in which central integrative regulation and peripheral effector mechanisms play complementary but distinct roles in OP progression and treatment, positioning neurotransmitter regulation and neuroinflammatory circuits as critical targets beyond bone-local mechanisms alone. From a clinical perspective, peripheral microbiota–neurotransmitter pathways may represent more accessible targets during active OP treatment, whereas modulation of central neuroendocrine and neuroinflammatory circuits may be particularly relevant for long-term prevention and risk reduction (Table 2).

### Chronic stress, the HPA axis, and a self-reinforcing loop

Chronic stress accelerates bone loss by persistently activating the HPA axis and exposing the body to prolonged glucocorticoid levels. Elevated cortisol suppresses osteoblast function, promotes bone resorption, and reshapes immune signaling. Stress hormones can also impair gut barrier integrity and alter gut physiology, creating conditions that favor dysbiosis. Dysbiosis, in turn, can influence stress pathways by producing neuroactive metabolites and inflammatory signals that reach the brain via humoral pathways and vagal afferents, reinforcing HPA activation and establishing a self-perpetuating cycle [54]. This self-reinforcing loop highlights an early intervention window in which stabilizing gut–brain communication may interrupt stress-driven skeletal deterioration before irreversible bone loss occurs. Translationally, stabilizing gut–brain communication may help attenuate stress-related skeletal deterioration. Preclinical work suggests that selected probiotics or prebiotics can partially normalize stress-associated HPA responses, although effects are context dependent and human evidence remains limited [55]. Accordingly, the most immediate implication is mechanistic: The HPA axis provides a plausible bridge linking stress, gut barrier dysfunction, neuroimmune activation, and bone remodeling [56]. Emerging delivery strategies, including nanocarrier approaches combined with exercise [57], further suggest that targeting stress circuitry may complement OP management, while requiring rigorous validation.

Notably, dysregulation of the gut–brain–bone axis mediated by chronic stress and HPA axis activation exhibits distinct patterns across major clinical subtypes of OP. In PMOP, estrogen deficiency sensitizes hypothalamic–pituitary signaling and disrupts gut barrier integrity, leading to microbiota dysbiosis, enhanced intestinal permeability, and elevated proinflammatory cytokines that converge on RANKL-dependent osteoclastogenesis [58,59]. In senile OP, age-related alterations in gut microbial diversity, reduced SCFA production, and progressive impairment of neuroendocrine resilience collectively promote a state of low-grade chronic stress, characterized by sustained cortisol exposure, neuroinflammation, and impaired osteoblast function [13,60]. By contrast, glucocorticoid-induced OP represents a more direct perturbation of this axis, in which prolonged exogenous glucocorticoid exposure drives persistent HPA axis activation, suppresses osteogenic signaling, compromises intestinal barrier function, and reshapes microbial metabolic output, thereby reinforcing a self-amplifying loop of gut dysbiosis, immune activation, and bone resorption [61,62]. Together, these observations highlight that while the gut–brain–bone axis constitutes a shared regulatory framework in OP, its pathological engagement is subtype specific, shaped by distinct neuroendocrine, immune, and microbial stressors, underscoring the importance of etiology-tailored microbiota-targeted and neuroendocrine-targeted interventions.

### Neurodegenerative diseases influence bone health through the gut–brain–bone axis

Neurodegenerative disorders progressively damage central neural circuits, resulting in cognitive decline, motor impairment, and muscle weakness. These alterations can secondarily disrupt bone metabolism by impairing neuroendocrine regulation and exacerbating systemic inflammation. Because central nervous system pathology often develops over prolonged periods and therapeutic delivery to the brain remains challenging, recent research has increasingly focused on peripheral systems and interorgan communication. Dysbiosis has been associated with mental and neurological disorders, and immune trafficking across tissues provides a mechanistic anchor for gut–immune–bone coupling. Single-cell studies in osteoarthritis have reported a gut-linked T cell subset in synovial tissue sharing T cell receptor features with intestinal T helper programs, supporting cross-tissue migration as part of a microbial–immune–bone axis [63]. In parallel, vagus-nerve-mediated microbiome signaling plays a critical role in mood and behavioral regulation, and microbiota transfer experiments have demonstrated that microbial communities derived from depression-like models can induce similar behavioral phenotypes in recipient animals. Neuromodulatory interventions, such as repetitive transcranial magnetic stimulation, have also been associated with restoration of intestinal microflora and improved barrier function in diet-associated cognitive dysfunction models, reflecting bidirectional brain–gut communication [64]. Importantly, this relationship is not unidirectional. The skeletal system may also influence brain health, as bone-derived cells and immune populations can release extracellular vesicles that regulate neural homeostasis. Under pathological conditions, vesicles carrying harmful cargo may reach the brain and aggravate neurodegenerative processes [65]. Osteocyte-derived extracellular vesicles have shown neuroprotective effects in animal studies, helping preserve cognition and skeletal integrity, yet this protective capacity appears to decline with aging, accompanied by a shift toward neuroinflammatory signaling profiles [66]. Together, the available evidence supports a reciprocal model in which neurodegeneration and OP reinforce one another through interconnected inflammatory, endocrine, and vesicle-mediated pathways, resulting in a progressive cycle that worsens both neural and skeletal outcomes. From a clinical standpoint, this model suggests that managing gut dysbiosis and systemic inflammation in neurodegenerative populations may have secondary benefits for skeletal preservation, highlighting the gut–brain–bone axis as a shared therapeutic landscape.

### Treg and Th17 cell balance as an immune hinge for gut–brain–bone coupling

**Fig. 3.**
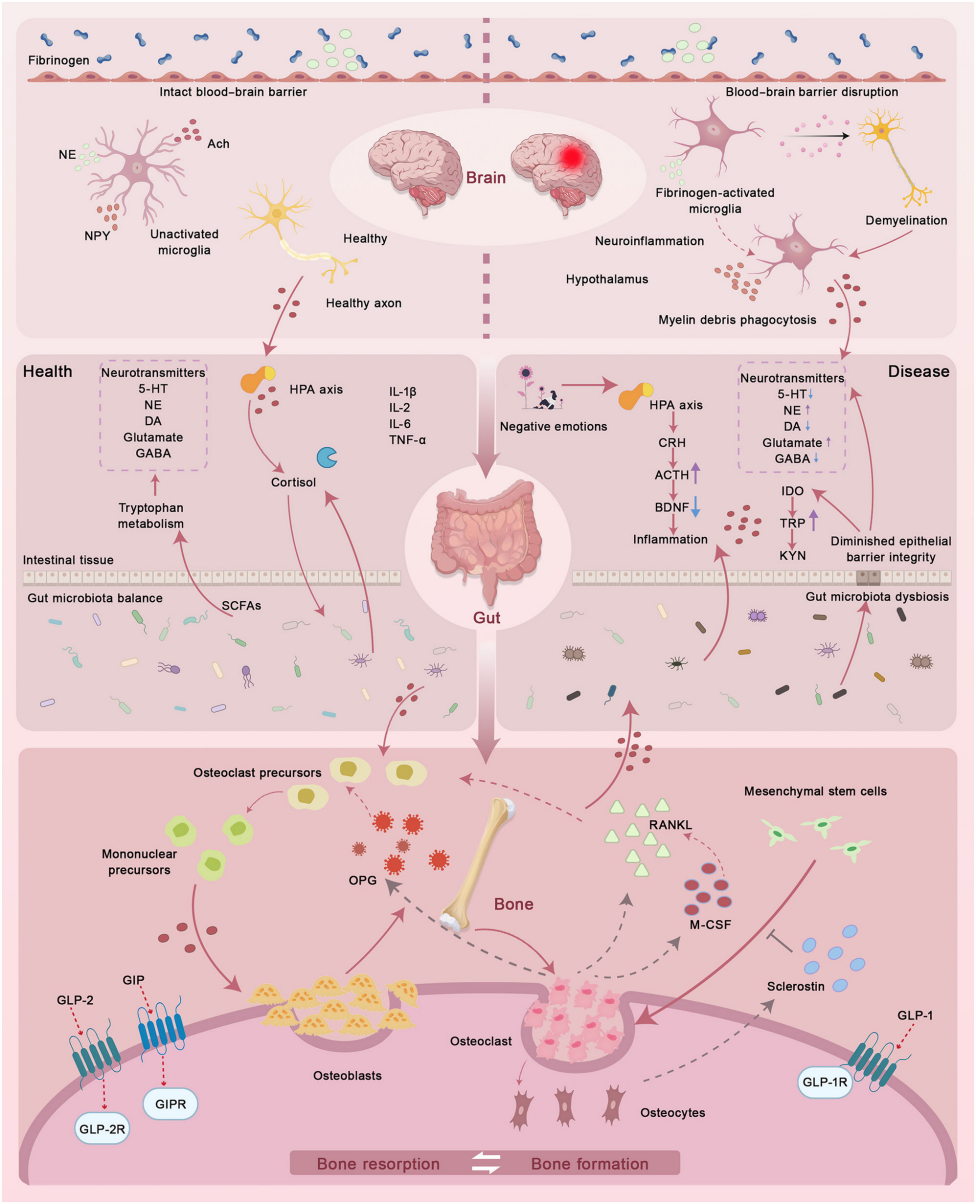
The figure illustrates the interplay between gut microbiota, neuroinflammation, and bone metabolism, summarizing the bidirectional interactions under physiological and pathological conditions. The top panel illustrates how an intact blood–brain barrier maintains neural homeostasis through regulated neurotransmitter signaling, including norepinephrine (NE), acetylcholine (Ach), and neuropeptide Y (NPY). In contrast, blood–brain barrier disruption triggers fibrinogen-mediated neuroinflammation, demyelination, and proinflammatory cytokine release. The central panel depicts gut-dysbiosis-induced activation of the hypothalamic–pituitary–adrenal axis, leading to elevated cortisol, altered tryptophan metabolism, and systemic inflammatory signaling that links gut imbalance to neural dysfunction and bone pathology. The bottom panel highlights gut-hormone-mediated regulation of bone remodeling, in which GLP-1, GLP-2, and GIP modulate osteoblast and osteoclast activity through OPG–RANKL–macrophage colony-stimulating factor (M-CSF) pathways, with GLP-1 receptor (GLP-1R) signaling further integrating gut–brain–bone cross-talk. GIPR, GIP receptor; GABA, γ-aminobutyric acid; BDNF, brain-derived neurotrophic factor; TRP, tryptophan; KYN, kynurenine; IDO, indoleamine 2,3-dioxygenase; DA, dopamine; CRH, corticotropin-releasing hormone; ACTH, adrenocorticotropic hormone.

As discussed above, osteoimmune mechanisms, particularly the balance between Treg and Th17 cells, contribute to OP by modulating osteoclastogenic signaling [67]. Within the gut–brain–bone axis, gutmicrobiota-driven immune programming plays a pivotal role by influencing neuroendocrine communication and central inflammatory tone, thereby indirectly regulating skeletal remodeling (Fig. 3) [68]. This immune hinge provides a unifying explanation for how microbiota-targeted interventions may exert multilevel effects, simultaneously dampening neuroinflammation, stabilizing endocrine signaling, and reducing osteoclastogenic pressure. Gut microbiota regulates bone homeostasis through interactions with innate immunity and pattern recognition receptors, with pathogenic bacteria inducing bone destruction and probiotic bacteria preventing bone loss, establishing a link between microbial ecology and bone health [69]. Microbiota-targeted approaches may therefore act as adjuncts by stabilizing barrier function, limiting antigen translocation, and shifting immune tone in a direction less permissive to bone loss. For example, *Lactobacillus acidophilus* has been reported in ovariectomized mice to improve trabecular and cortical microarchitecture and increase bone mineral density [70].

### Microbiota regulation of neuroendocrine hormones and indirect effects on bone quality

In postmenopausal women, reduced microbial richness with compositional shifts has been reported, including lower *Firmicutes*, *Butyricicoccus*, and *Bifidobacterium,* and higher *Bacteroides*, *Proteus*, and *Campylobacter*, alongside systemic signatures consistent with barrier dysfunction [61]. Beyond immune pathways as described above, gut microbiota can influence bone quality by modulating neuroendocrine hormone production and signaling (Fig. 3) [71]. Neuroendocrine links have also been observed in animal models. In Dahl salt-sensitive rats, a high-salt diet altered gut microbial composition and increased hypothalamic agouti-related peptide neuron activity, which was associated with elevated levels of the bone resorption marker C-terminal telopeptide of type I collagen. Glucocorticoid biology represents another neuroendocrine route. Excess cortisol or exogenous glucocorticoids impair calcium handling and osteoblast survival and can increase bone resorption, contributing to OP risk [61]. Gut-derived serotonin provides an additional mechanism. The microbiota can regulate enterochromaffin TPH1 and peripheral 5-hydroxytryptamine (5-HT) production. Peripheral 5-HT has been linked to enhanced RANKL-mediated osteoclastogenesis, and blocking TPH1 prevents bone loss in ovariectomized mice [72]. The microbiota has also been implicated in modulating insulin-like growth factor 1 and parathyroid hormone signaling relevant to skeletal dynamics [71]. Overall, microbiota-directed strategies should be evaluated not only for their direct effects on skeletal outcomes but also for their broader endocrine and neuroimmune consequences in well-designed clinical trials.

### Synergy between dietary regulation and neurotransmitter-related modulators

Dietary exposures can shape skeletal outcomes through microbiota-linked and neuroendocrine routes [73]. These changes were accompanied by trabecular bone deterioration, decreased microbial richness, and enrichment of specific taxa, including Defluviitaleaceae UCG-011, *Erysipelatoclostridium*, and Ruminococcaceae UCG-009. These findings support the concept that diet-induced dysbiosis is closely coupled with endocrine alterations relevant to bone metabolism. Protein nutrition, particularly whey protein, has also been reported to influence bone metabolism via microbiota modulation. For example, stimulation of *Bifidobacterium animalis* and *Lactobacillus* strains has been associated with improved osteogenic signaling and reduced circulating indices of bone resorption, including C-terminal telopeptide and parathyroid hormone, in experimental settings [74]. These microbial-mediated effects on bone may further involve microbial metabolites and amino acid pathways, including tryptophan metabolism, which can intersect with neurotransmitter biology and bone turnover, potentially contributing to increased bone resorption in OP [75,76]. Collectively, these findings suggest that dietary interventions may influence gut–brain–bone crosstalk, providing a potential avenue to modulate bone metabolism.

Probiotic interventions may complement neuromodulatory approaches by modulating gut–brain communication. In osteoporotic rat models, *Bifidobacterium longum* has been reported to increase osteogenic gene expression [77], improve bone mass and density, and attenuate inflammatory signatures, suggesting immunometabolic support for skeletal preservation [78]. More broadly, gut-microbiota-derived metabolites can communicate with the brain via vagal pathways, offering a mechanistic rationale for combining microbiota-based interventions with neuromodulatory therapies in OP and related bone disorders [19].

## Therapeutic Strategies and Clinical Translation

### FMT as a system-level option for OP

FMT is an emerging therapeutic strategy aimed at restoring gut microbial homeostasis and shows considerable potential for the treatment of OP (Table 3) [79]. Conceptually, its therapeutic rationale involves a coordinated sequence of reshaping microbial community structure, reinforcing gut barrier integrity, and dampening systemic inflammation, thereby rebalancing osteoimmune signaling. From a peripheral regulatory perspective, these effects primarily originate in the gut microenvironment and immune compartment, where microbial metabolites and barrier integrity directly influence bone remodeling. In experimental models of OP, FMT has been shown to reduce osteoclastogenesis while enhancing osteoblast function, collectively contributing to skeletal preservation [80].

**Table 3.** Treatment strategies and clinical transformation

Strategy	Related treatment mechanisms	Effects	Clinical application prospects	Future challenges
FMT	Improves the composition of the intestinal microbiota and increases SCFAs	Enhances osteoblast function and inhibits osteoclast activity	Providing new options for the prevention and treatment of OP	More research is needed to verify the personalized treatment strategies, donor screening criteria, safety, and long-term effects of FMT
	Improves intestinal barrier integrity	Improves intestinal barrier function and reduces bone resorption		
	Inhibits the release of osteoclastogenic cytokines such as TNF-α and IL-1β	Regulates the intestinal immune environment, inhibits inflammatory response, and reduces the occurrence of OP		
	Restores the balance of intestinal metabolites (such as bile acids, indole derivatives, vitamins D, C, and K)	Promotes bone formation and inhibits bone resorption		
Probiotics	Modulates gut microbiota and osteoblast metabolic pathways	Improves calcium absorption, increases bone density and improves trabecular bone structure	It has shown great therapeutic potential in regulating the gut microbiota and improving bone metabolism	Long-term safety, specific mechanisms, and efficacy differences among different strains still require further study
	Promotes the production of SCFAs	Regulates the intestinal acid-base environment and inhibits osteoclast activity		
	Promotes the growth of beneficial bacteria, enhances the production of SCFAs, and improves mineral absorption	Lowers intestinal pH, improves calcium and magnesium absorption, and optimizes BMD		
Melatonin	Improves intestinal barrier function and regulates the composition of intestinal microbiota	Reduces the formation of harmful metabolites such as TMAO and protects bone health	Fewer side effects and longer-lasting efficacy	Supervision and standardization of clinical applications still need to be further improved
Vitamin K2	Restores the abundance of *Lactobacillus*, *Bifidobacterium*, and *Clostridium* and alleviates oxidative stress and inflammatory responses	Improves BMD		
Strontium ranelate	Modulates the gut microbiota, changes its composition, and enhances BMD	Promotes osteoblastogenesis and inhibits osteoclast activity		Impact on the gut microbiome has not been well studied
Natural plant extracts (such as Korean red ginseng)	Modulates the composition of the gut microbiota	Markedly improves glucocorticoid-induced OP in a mouse model	Used in combination with traditional medicines to enhance therapeutic effects and reduce side effects	Limited effects on gut barrier and immune cell populations
Ketamine and its derivatives	Change the structure of the intestinal microbiota and regulate SCFAs and related metabolites	Increase BMD and slow down bone loss	Combination with prebiotics provides a new direction for the treatment of OP	The exact molecular mechanism and long-term safety need to be verified

Mechanistically, these benefits may involve strengthened tight-junction architecture, including increased expression of barrier-related proteins, which can reduce intestinal permeability and limit endotoxin translocation that otherwise sustains inflammation and accelerates bone resorption [81]. Microbial metabolic remodeling provides an additional layer of benefit. FMT has been linked to enhanced production of SCFAs, which participate in immune regulation and support bone remodeling (Fig. 3). SCFAs are reported to promote osteoblast activity while constraining osteoclast differentiation; this aligns microbial metabolism with skeletal homeostasis. Beyond these peripheral mechanisms, emerging evidence suggests that FMT-induced microbial and metabolic changes may also engage central regulatory pathways along the gut–brain–bone axis. SCFAs and gut-derived immune signals can modulate vagal afferents and hypothalamic neuroendocrine circuits, thereby indirectly influencing sympathetic tone and bone turnover [82]. Such central regulatory engagement may become particularly relevant during treatment phases, where systemic metabolic and neuroendocrine reprogramming contributes to sustained skeletal benefits beyond local gut effects. From this perspective, FMT represents a route to improve bone health by jointly tuning microbiome composition and functional metabolic output [83]. Beyond estrogen deficiency settings, animal studies also suggest protective effects in glucocorticoid-associated OP, consistent with barrier repair and inflammatory control that counteract medication-related skeletal damage [84]. Despite these promising findings, the clinical translation of FMT remains challenging. Key obstacles include donor screening, procedural standardization, microbial strain viability during administration, and the development of personalized treatment protocols. Well-designed clinical trials are required to establish safety, efficacy, and responder characteristics, as well as to determine how FMT may be optimally integrated with other interventions, such as exercise or pharmacological therapies.

#### Clinical validation and challenges of probiotics, prebiotics, and postbiotics

**Fig. 4.**
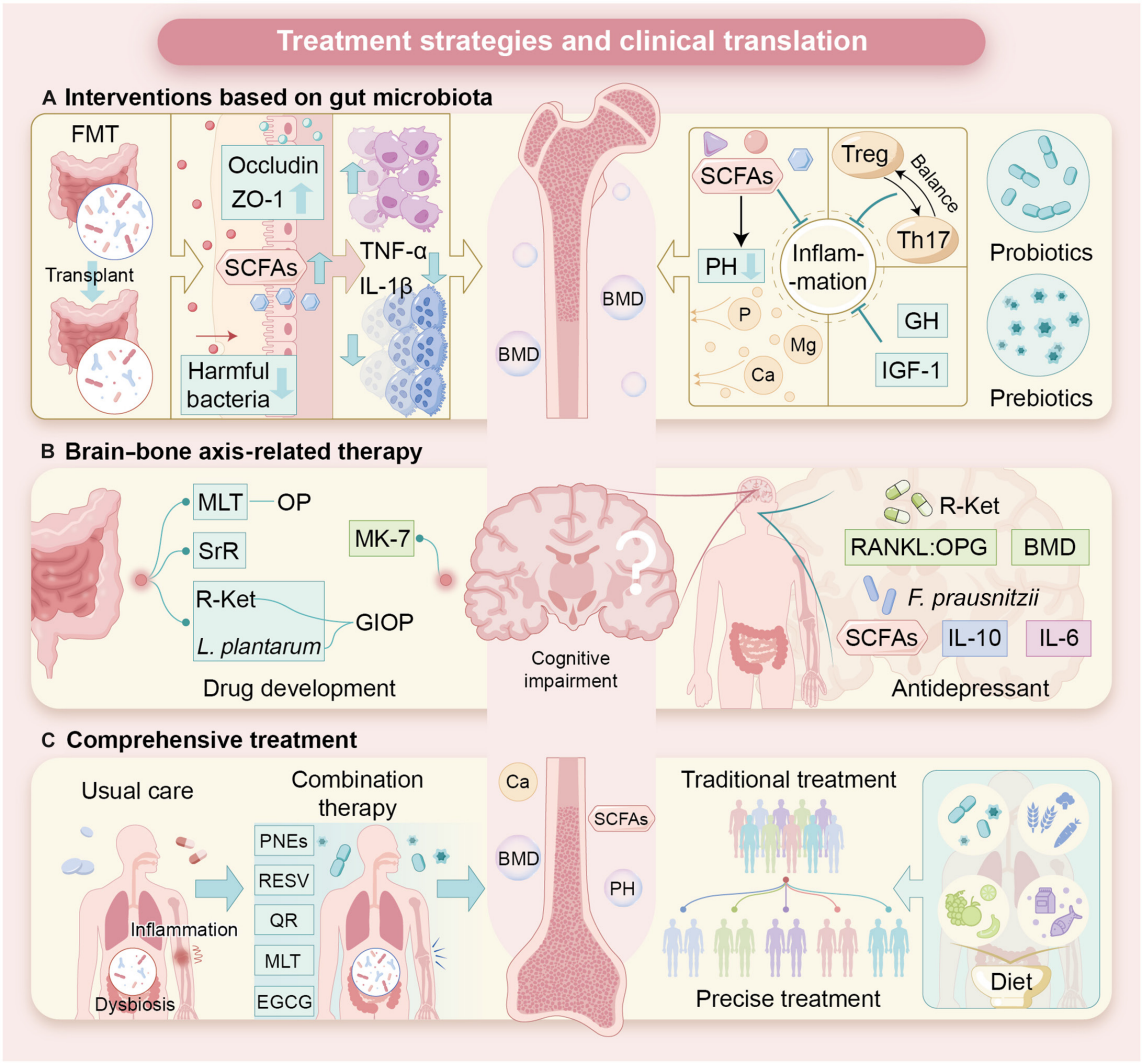
This figure illustrates treatment strategies and clinical translation in OP and bone metabolism. (A) Approaches to restore microbial balance, including FMT, which enhances intestinal barrier integrity by up-regulating tight junction proteins occludin and ZO-1 and suppresses proinflammatory cytokines such as TNF-α and IL-1β. Gut-microbiota-derived SCFAs support Treg/Th17 cell balance and mineral metabolism, while probiotics and prebiotics contribute to improved bone mineral density (BMD). IGF-1, insulin-like growth factor 1. (B) Brain–bone axis-targeted interventions, including R-ketoglutarate (R-Ket), MK-7, melatonin, and *Lactobacillus* plantarum, which regulate RANKL and OPG signaling and modulate inflammatory cytokines to support both neural and bone functions. MLT, melatonin; SrR, strontium ranelate. (C) Integrated management strategies combining targeted therapies and dietary interventions to alleviate gut dysbiosis, inflammation, and cognitive impairment. PH, parathyroid hormone; GH, growth hormone; GIOP, glucocorticoid-induced osteoporosis; PNEs, polyphenols; RESV, resveratrol; QR, quercetin; EGCG, epigallocatechin gallate.

Probiotics, prebiotics, and postbiotics have attracted increasing attention for their potential to restore gut microbiota homeostasis and improve bone health. These microbiota-based interventions are now considered as adjuncts or potential alternatives to conventional OP therapies. Their therapeutic effects are predominantly mediated through peripheral regulatory mechanisms, including modulation of gut immunity, microbial metabolite production, and intestinal mineral absorption. Animal studies have revealed that *B. longum* and *L. rhamnosus* can enhance osteogenic gene expression and help gain bone mass (Fig. 4). The beneficial effects associated with *Bifidobacterium* are often attributed to systemic immunomodulation, including the activation of regulatory immune pathways that suppress osteoclast-mediated bone resorption. In contrast, *Lactobacillus* strains more frequently exert localized anti-inflammatory effects within the gut, reducing proinflammatory mediators that indirectly shift skeletal remodeling toward resorption. These strain-specific effects highlight functional heterogeneity within peripheral regulatory pathways of the gut–bone axis (Table 3). At the same time, accumulating evidence suggests that certain probiotic-derived metabolites may also influence central regulatory circuits. For example, microbial modulation of tryptophan metabolism and SCFA signaling can affect hypothalamic appetite regulation, stress responsiveness, and autonomic output, all of which have downstream consequences for bone metabolism. Compared with FMT, however, these central effects appear more limited and indirect, positioning probiotics and related interventions primarily as peripheral modulators during both disease progression and treatment. Prebiotics, such as inulin-type substrates, oligosaccharides, and resistant starch, may reshape the microbial environment and support mineral absorption, thereby contributing to bone protection (Fig. 4). When administered together, synbiotics may improve probiotic viability and metabolic activity, thereby reinforcing microbial functions that support mineral utilization and osteoimmune homeostasis [85]. However, direct comparative evidence between synbiotic formulations and single-component interventions remains limited. Well-designed, large-scale clinical trials are therefore required to establish reproducibility and generalizability of these findings. Differences in strain selection, dosing schedules, formulation, and baseline host context likely contribute to variability. These results highlight that microbial responses are host dependent and shaped by genetics, aging biology, and baseline health, making uniform application across populations unreliable. While probiotics are generally regarded as safe, clinical utility requires clearer criteria for strain choice, treatment duration, target skeletal sites, and responder stratification. Emerging adjuncts, including polyphenol-rich natural compounds, may also shape microbial diversity and oxidative and inflammatory status, but their long-term integration with microbiota-based interventions requires further validation.

#### Drug development targeting neurotransmitters and hormones

**Fig. 5.**
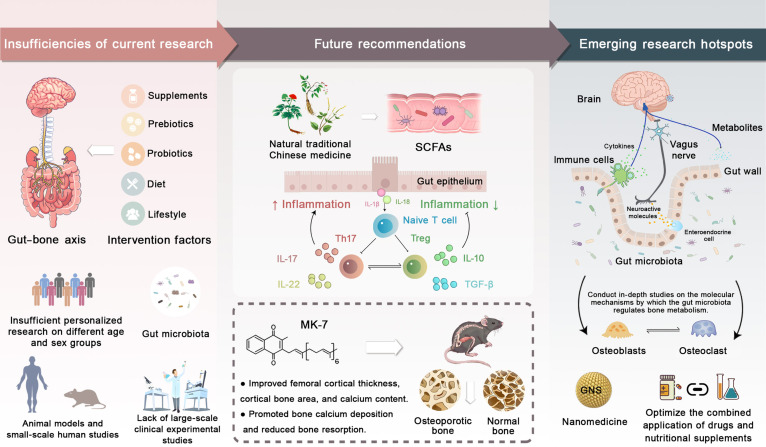
Current research, future recommendations, and emerging research hotspots in gut microbiota and bone health. This figure summarizes current limitations, future directions, and emerging research priorities in the role of gut microbiota in bone metabolism and OP. The left panel highlights existing gaps, including limited personalization across age and sex, insufficient large-scale clinical trials, and heavy reliance on animal models. The middle panel outlines future strategies including the application of natural traditional Chinese medicine, SCFAs, and MK-7 to modulate inflammation, promote Treg/Th17 cell balance, enhance calcium deposition, and reduce bone resorption. The right panel illustrates emerging research hotspots centered on the gut–brain–bone axis, emphasizing vagus nerve signaling, immune regulation, molecular-level mechanisms, and optimized drug–nutrient combination therapies for OP prevention.

Recent studies have demonstrated that the gut microbiota plays a critical role in bone metabolism (Fig. 5). Targeting the gut microbiome may provide new treatment options for patients with OP. Within this framework, the gut–brain–bone axis has emerged as a key integrative pathway, in which gut-derived metabolites influence central nervous system activity, thereby modulating bone remodeling. Importantly, this axis involves both peripheral regulatory mechanisms originating in the gut and immune system, as well as central regulatory mechanisms mediated by neuroendocrine and neurotransmitter signaling. Emerging research indicates that gut metabolites can act on the brain, which, in turn, influences osteoclast-mediated bone resorption through autonomic and hormonal outputs. Neurotransmitters and hormones such as serotonin, dopamine, and melatonin are now recognized as critical mediators linking gut microbial activity with central regulation of bone metabolism [86]. Peripheral serotonin, largely produced in the gut, primarily promotes bone resorption, whereas centrally derived serotonin exerts distinct regulatory effects through hypothalamic pathways, highlighting a clear division between peripheral and central regulatory roles within the same molecular system [87]. From a peripheral perspective, melatonin is an endogenous hormone that modulates circadian rhythms and benefits bone health by improving gut barrier function and modulating the gut microbiome [88]. Taking melatonin supplements can help restore microbiome diversity, boost beneficial bacteria such as *Allobaculum* and *Parasutterella,* and increase the production of SCFAs. In addition, melatonin regulates macrophage polarization, leading to reduced inflammation and improved gut homeostasis. Concurrently, melatonin acts as a central regulator through circadian and neuroendocrine pathways, influencing sympathetic activity and systemic metabolic rhythms that indirectly shape bone turnover. These dual actions suggest its potential as a comprehensive therapeutic agent for OP engaging both peripheral and central regulatory mechanisms. Vitamin K2 (menaquinone-7 [MK-7]), a metabolite produced by gut bacteria, also exhibits therapeutic potential in bone-related disorders. MK-7 contributes to skeletal health and prevents ectopic calcification in cardiovascular and neural tissues. Evidence indicates that MK-7 can restore microbial balance, reduce oxidative stress, and promote neuronal health, highlighting its value as a multitarget therapeutic agent for diseases associated with gut dysbiosis [89]. Strontium ranelate, a clinically used antiosteoporotic agent, enhances osteoblast activity while inhibiting osteoclast function. A recent study has shown that strontium ranelate alters the gut microbiome by increasing the abundance of beneficial bacteria such as *Ruminococcus albus* and *Akkermansia*. This alteration in the gut microbiome results in improved bone metabolism and higher levels of metabolites such as lycopene and pimelic acid. The results suggest that strontium ranelate might be utilized to modulate the gut microbiome–bone axis, providing a novel treatment for OP (Fig. 3) [90]. These effects are predominantly mediated through peripheral gut–bone pathways; however, secondary influences on gut–brain signaling cannot be excluded. Natural products, such as ginseng extract, have shown protective effects in models of glucocorticoid-induced OP. Ginseng markedly attenuates glucocorticoid-induced bone loss by increasing gut microbial diversity. Research shows that neurotransmitter- or hormone-targeted interventions can help improve bone health by modifying gut microbiota (Fig. 5) [91]. In summary, drugs targeting the gut–bone axis show considerable promise for the treatment of OP. Melatonin, vitamin K2, and strontium ranelate therefore represent promising candidates within an emerging class of microbiota-responsive therapeutics for OP (Fig. 5).

#### Emerging applications of antidepressants in bone metabolism regulation

The interplay among mood-related pharmacotherapies, gut microbiota, and bone remodeling has attracted increasing scientific attention. This interaction is particularly relevant to the central regulatory dimension of the gut–brain–bone axis, as antidepressants primarily target neural and neuroendocrine pathways. Experimental evidence suggests that certain antidepressants, including ketamine-related compounds, can reshape gut microbial composition and metabolic activity in ways associated with improved bone outcomes (Fig. 3) [92]. Arketamine, a ketamine enantiomer with superior antidepressant efficacy, has been shown to enhance bone mineral density in animal models of chronic stress and OP (Table 3) [93]. Mechanistic studies suggest that arketamine modulates the gut microbiome by increasing microbial diversity, enriching beneficial bacterial taxa, and elevating levels of SCFAs. These microbiota-mediated changes represent a peripheral regulatory component that complements the primary central actions of antidepressants on neural circuits and stress-related hormonal axes. Moreover, arketamine may influence bone metabolism through the regulation of key signaling pathways. In particular, the RANKL/OPG axis, a central regulator of osteoclastogenesis, has been implicated in this process. Concurrently, psychobiotic candidates such as *F. prausnitzii* have been proposed as microbial adjuncts capable of supporting both neurobehavioral and skeletal outcomes, potentially through enhanced SCFA production and attenuation of inflammatory responses [94]. Together, these findings support a bidirectional model in which central neuroendocrine regulation and peripheral-microbiota-driven immune signaling are jointly rebalanced. This integrative strategy offers a translational hypothesis for addressing comorbid depression and OP.

#### Precision medicine based on individual gut microbiota features

Given strong interindividual variability, OP management may benefit from precision strategies that align interventions with genetic background, microbiome composition, and lifestyle context. Such variability may influence both peripheral microbial responsiveness and central neuroendocrine sensitivity within the gut–brain–bone axis. Dietary patterns are consistently linked to microbial ecology and metabolic outputs that can influence bone remodeling. Diets enriched in plant-derived foods and unsaturated fats are generally associated with more favorable metabolic and inflammatory states, whereas energy-dense diets rich in sugar and saturated fat are linked to dysbiosis and bone vulnerability [95]. Microbiota-supportive foods and prebiotic compounds, including arabinoxylan-derived oligosaccharides, have been proposed to promote the growth of beneficial taxa such as *Bifidobacterium* and *Lactobacillus*, potentially complementing pharmacological therapies and supporting bone health [96]. Beyond nutritional modulation, phage-based approaches have been proposed as tools to reshape microbial communities with greater specificity, potentially reducing reliance on broad-spectrum antibiotics and addressing antimicrobial resistance concerns [97]. Ultimately, the integration of genomic, microbiome, and lifestyle data may enable individualized prevention and treatment strategies that enhance therapeutic efficacy, minimize adverse effects, and better identify responder subgroups for microbiota-targeted interventions.

## Conclusion and Outlook

Accumulating evidence supports that the gut microbiota contributes substantially to bone remodeling and represents a promising, albeit still exploratory, target for OP prevention and therapy. As described above, perturbations in microbial ecology may coincide with impaired intestinal barrier integrity, low-grade inflammation, and altered gut–brain communication, collectively shifting skeletal homeostasis toward bone loss. Beyond bone-local signaling, emerging data highlight the gut–brain–bone axis as an integrative regulatory network linking microbial metabolites, immune mediators, and neuroendocrine pathways to osteoblast and osteoclast activity. In particular, gut-derived SCFAs, hormones, and stress-responsive signaling provide mechanistic routes through which the microbiome can influence skeletal remodeling. Estrogen deficiency further heightens susceptibility to these systemic perturbations, amplifying microbiome-associated immune and neuroendocrine alterations that are especially relevant in PMOP.

Despite rapid advances, translation into routine clinical practice remains limited. Key challenges include the heterogeneity of microbiome profiles across populations, inconsistent trial designs, variable strain- and dose-dependent effects, and incomplete control of confounders such as diet, medications, comorbidities, and baseline fracture risk. Safety considerations are particularly important for interventions involving live organisms or community transfer, necessitating standardized manufacturing, donor screening, long-term surveillance, and regulatory clarity. Future trials should be adequately powered and of sufficient duration, incorporate harmonized skeletal end points including fracture-related outcomes when feasible, and adopt responder stratification based on baseline microbiome features and host characteristics. Looking ahead, the integration of multiomics approaches with systems biology and artificial-intelligence-assisted modeling will be essential to advance from association to causality, identify actionable microbial functions and biomarkers, and inform individualized therapeutic strategies. A precision-medicine framework that combines microbiome profiles with dietary patterns, lifestyle factors, and host genomic context may ultimately enable safer, more effective, and more personalized microbiota modulation for OP.
